# Identification of Ictal Tachycardia in Focal Motor- and Non-Motor Seizures by Means of a Wearable PPG Sensor

**DOI:** 10.3390/s21186017

**Published:** 2021-09-08

**Authors:** Martin Glasstetter, Sebastian Böttcher, Nicolas Zabler, Nino Epitashvili, Matthias Dümpelmann, Mark P. Richardson, Andreas Schulze-Bonhage

**Affiliations:** 1 Epilepsy Center, Department of Neurosurgery, Medical Center—University of Freiburg, 79106 Freiburg im Breisgau, Germany; sebastian.boettcher@uniklinik-freiburg.de (S.B.); nicolas.zabler@uniklinik-freiburg.de (N.Z.); nino.epitashvili@uniklinik-freiburg.de (N.E.); matthias.duempelmann@uniklinik-freiburg.de (M.D.); andreas.schulze-bonhage@uniklinik-freiburg.de (A.S.-B.); 2 Division of Neuroscience, Institute of Psychiatry, Psychology & Neuroscience King’s College London, London SE5 9RT, UK; mark.richardson@kcl.ac.uk

**Keywords:** photoplethysmography (PPG), heart rate, signal quality, motor and non-motor seizures, ictal tachycardia, wearable device

## Abstract

Photoplethysmography (PPG) as an additional biosignal for a seizure detector has been underutilized so far, which is possibly due to its susceptibility to motion artifacts. We investigated 62 focal seizures from 28 patients with electrocardiography-based evidence of ictal tachycardia (IT). Seizures were divided into subgroups: those without epileptic movements and those with epileptic movements not affecting and affecting the extremities. PPG-based heart rate (HR) derived from a wrist-worn device was calculated for sections with high signal quality, which were identified using spectral entropy. Overall, IT based on PPG was identified in 37 of 62 (60%) seizures (9/19, 7/8, and 21/35 in the three groups, respectively) and could be found prior to the onset of epileptic movements affecting the extremities in 14/21 seizures. In 30/37 seizures, PPG-based IT was in good temporal agreement (<10 s) with ECG-based IT, with an average delay of 5.0 s relative to EEG onset. In summary, we observed that the identification of IT by means of a wearable PPG sensor is possible not only for non-motor seizures but also in motor seizures, which is due to the early manifestation of IT in a relevant subset of focal seizures. However, both spontaneous and epileptic movements can impair PPG-based seizure detection.

## 1. Introduction

Wearable devices are increasingly deployed for applications in the home environment to assist people with epilepsy in their daily life, which is applicable for example as an early warning system for an upcoming epileptic seizure or for the retrospective detection/analysis of epileptic seizures in order to optimize treatment strategies.

Biosignals acquired by means of electroencephalography (EEG) are considered as the standard for the detection of epileptic seizures. However, EEG is routinely used in a hospital setting due to problems in the long-term stability of signals and due to the stigmatization associated with scalp electrode [1,2,3].

Non-EEG biosignals can assess aspects of seizure manifestations and can be recorded in everyday life with the aid of wearable devices. These biosignals include e.g., accelerometry (ACC), electrodermal activity (EDA), body temperature, electromyography, or photoplethysmography (PPG). Both individual signal types and their combinations have been used to detect epileptic seizures [4,5]. So far, mostly bilateral tonic-clonic seizures and seizures with major motor components have been studied as to their detectability using non-EEG signals from wearables [6,7,8,9,10,11,12,13]. Thus, the vast majority of wearable devices used in epileptic seizure detection are also tailored to the detection of these convulsive seizures [14,15]. Thereby, motion artifacts are a major source of bad signal quality, and it has been the common understanding that especially during the ictal phase, PPG signals are not beneficial for seizure detection [16]. However, it has been shown that the integration of autonomic signals in the detection of convulsive seizures, based mainly on ECG, can improve the performance of seizure detection [17,18,19,20,21,22,23].

The work presented here does not target the development of a seizure detection methodology. Instead, we here focus on investigating the quality and usefulness of the PPG signal during seizures with a clear autonomic component, consisting of an increase of the heart rate (HR) during the ictal phase [24], which are both regarded as a priori knowledge here. Studies comparing ictal tachycardia (IT) with scalp and intracranial EEG recordings have shown that a relevant subgroup of patients with focal seizures has an accompanying HR increase, frequently as an initial or early clinical seizure manifestation [25,26,27,28,29,30,31,32].

For reasons of comfort, wearables often do not use ECG recordings acquired via skin electrodes but rather PPG signals to assess heart rate. PPG is an optical measurement method for recording the change in blood volume over time in peripheral tissue through the use of LEDs and measured reflection [33]. Other promising opto-electronic methods have been investigated in recent advances in the field of wearable sensors [34,35]; however, these are not yet available for clinical use. To study the value of wearables for the identification of autonomic seizure components, we here perform a comparison of PPG signals recorded using a wearable designed for scientific studies with video-EEG long-term recordings including a one-channel ECG. As the reliability of recordings of autonomic phenomena may depend on the presence of simultaneous movements [16,36,37], we studied three subgroups of patients with seizures displaying an autonomic component:

- **Group 1:** Seizures with IT and without epileptic movements (non-motor)
- **Group 2:** Seizures with IT and with epileptic motor symptoms not affecting the extremities, including oral automatisms or eye blinking (motor, non-relevant)
- **Group 3:** Seizures with IT and with epileptic movements involving arms or legs (motor, relevant)

The groups were divided based on clinical expert annotations of epileptic movements, as described in the next section. Two threshold crossings were used to define IT, and the respective time points were related to the EEG onset of the seizure. ECG and PPG signals were compared with regard to the timing of identification of IT. Our primary aim was to distinguish patient subgroups in whom an ambulatory identification of ictal tachycardia based on PPG is feasible for seizure detection. To the best of our knowledge, this is the first study to provide a comparison between PPG- and ECG-based identification of ictal tachycardia while also analyzing the effect of movements on PPG signal quality during IT.

## 2. Materials and Methods

### 2.1. Data Acquisition

All signals were acquired in the epilepsy monitoring unit of the Freiburg epilepsy center during continuous video-EEG monitoring, where patients usually stay for a week, are largely constrained to their bed, and are only able to walk short distances to the bathroom. This means that they were immobile for longer than usual compared to their everyday life but not completely inactive. The ECG signal was recorded with bipolar electrodes placed below the right clavicle and above the left costal arch at a sampling frequency of 250 Hz. In addition to the routinely recorded EEG and ECG, patients wore the wristband Empatica E4 device (Empatica SRL, Via Enrico Stendhal 36, 20144 Milano (MI), Italy), which, in addition to ACC, EDA, and skin temperature also captures HR by means of PPG signals. ACC and PPG signals were recorded with a sampling frequency of 32 Hz and 64 Hz, respectively. The data collected by the wearable device were synchronized to the ECG signals recorded by the video-EEG system [38]. The PPG sensor mainly consists of a green LED and a photodetector. It is a reflective PPG sensor; thus, the signal is produced by measuring the amount of reflection of the light emitted into the skin, which changes with the volume of blood in the tissue. Therefore, the signal of reflected light shows the pulsing blood volume from which the heart rate can be determined. Note that the Empatica E4 device produces a preprocessed and optimized signal for the detection of the pulse wave, and not the raw PPG sensor response.

**Figure 1:**
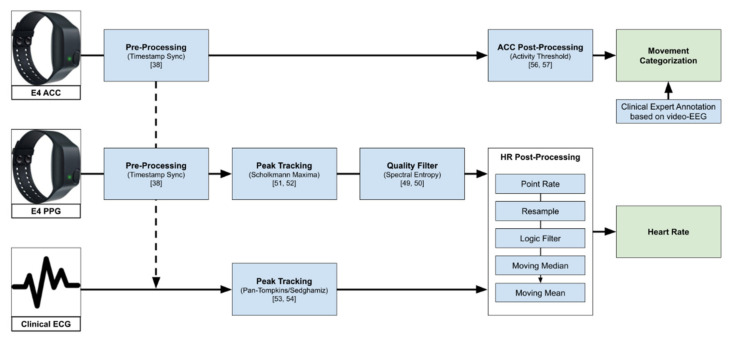
Overview of the data processing pipeline, from raw data to heart rate used in the further analysis, and the movement categorization based on ACC (see Section 3.4). The logic filter here describes the step of filtering point rate values that are not in the range of 40–180 bpm or changed by more than 20% from one value to the next.

Figure 1 gives an overview of the data processing pipeline explained further in the rest of this chapter.

For the purpose of this study, only seizures with evidence for an ictal HR increase, reflecting effects on the autonomic nervous system, were included. This was assessed quantitatively by a clinical expert (NE) using the simultaneously recorded ECG signal. Furthermore, only seizures with a minimum seizure duration of 10 s and sufficient ECG signal quality were analyzed. A maximum of five representative seizures per patient were selected to minimize potential bias. Two seizures were discarded due to their significantly longer duration of about 22 min and 45 min, respectively, as compared to all other seizures in the dataset.

This resulted in a total of 62 seizures from 28 patients with a seizure-related increase in HR. Detailed information on patient demographics is summarized in Table A1 in the Appendix A. The ictal phase was defined as the interval between EEG onset and offset of a seizure, as marked by clinical experts through visual inspection. The corresponding ECG, PPG and ACC signals were only analyzed in the ictal and peri-ictal phases, i.e., only in the immediate interval around and including each seizure, lasting from 60 s before EEG seizure onset to 30 s after EEG seizure offset. All signal modalities were processed using MATLAB R2020b (MathWorks, Natick, MA, USA).

The respective data segments were divided in a baseline interval (from −60 s to −30 s prior to EEG onset), a preictal phase (from −30 s prior to EEG onset), the ictal phase, and a postictal phase (30 s after EEG offset).

The study was performed according to Good Clinical Practice as part of the RADAR-CNS project (www.radar-cns.org, accessed on 3 September 2021), with ethical permission (ethics committee vote 538/16); each patient gave informed consent for data acquisition, storage, and analysis.

### 2.2. PPG Signal Quality Assessment

**Figure 2:**
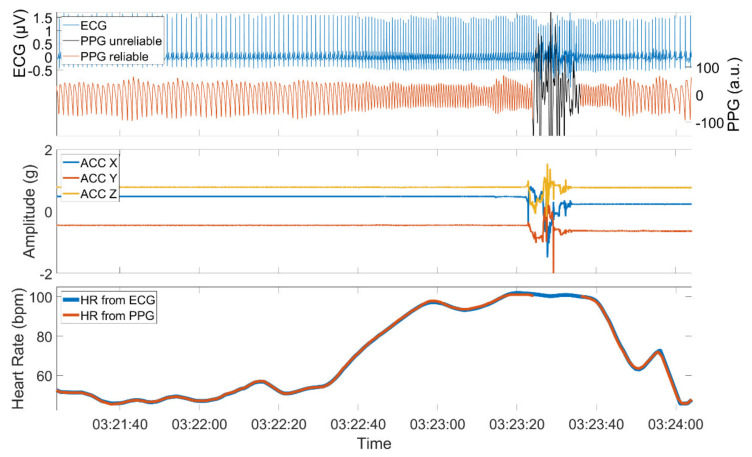
PPG and ECG signal (upper panel), the simultaneously recorded ACC signal (middle panel), and the derived PPG and ECG heart rate (lower panel). The PPG signal is composed of sections with high quality when the patient is at rest and sections of low quality when the patient is active. The PPG signal was automatically assessed using time-resolved spectral entropy to identify intervals containing artifacts.

The quality of a PPG signal is negatively affected by external influences such as body motion or ambient light that is captured by the sensor. Additionally, physical barriers that hinder the proper emission, absorption, or reflection of the used light sources can influence the signal quality. High-quality PPG signals show a stereotypical quasi-periodic and a positively skewed waveform between consecutive minima [39]. This waveform can be distorted when affected by one of the aforementioned reasons. Figure 2 shows an example of a PPG signal with sections of high and low quality, the simultaneously recorded ACC signal, and the derived HRs from both PPG and ECG.

Several promising methods have been deployed with the common approach to separate the signal from interference caused by motion in order to restore the evaluable part of the signal [40,41,42,43,44].

A different approach, chosen in this study, would simply discard the low-quality parts of the PPG signal [45,46,47,48]. Taking the quasi-periodic nature of the high-quality PPG signal as a basis, the power should be concentrated within a very limited number of frequency components. In contrast to this, a noisy signal exhibits a spectrum with a more even distribution of signal power. A metric that exploits these facts and which has been shown to be useful to distinguish between low and high-quality PPG signals [49] is the so-called spectral entropy (*SE*) given by:

(1) $$SE=-\frac{\sum_{f=f_{1}}^{f2}\hat{S}\left(f\right)\;\cdot \;log_{2}\left(\hat{S}\left(f\right)\right)}{log_{2}\left(N\right)}$$

where $\hat{S}\left(f\right)$ is the periodogram, which gives an estimate of the power spectral density PSD $S\left(f\right)$. Note that $\hat{S}\left(f\right)$ is normalized to one in the frequency interval $\left[f_{1},f_{2}\right]$ before calculating *SE* and that the denominator assures 0≤SE≤1 with N being the number of frequency bins in the interval $\left[f_{1},f_{2}\right]$. The quantity *SE* is equal to 0 for a signal composed of a single spectral component and equal to 1 for a signal with a constant spectrum. The upper and lower frequency was set to *f*_1_ = 0.1 Hz and *f*_2_ = 5 Hz, respectively, as we expected the signal fundamental frequency and higher harmonics to lie within this range. The periodogram was calculated in a moving time window with a length of four seconds and overlap of 3.75 s. In order to mitigate the effect of broadband spectral leakage, a Tukey window was applied before the calculation of the periodogram [50].

A threshold of *SE* = 0.72 was empirically found to be reasonable in order to separate reliable PPG sections from PPG sections of insufficient quality. A section with a calculated value *SE* below that threshold was considered for further evaluation; otherwise, it was assessed as unreliable.

### 2.3. PPG and ECG Peak Tracker

The Empatica E4 device provides an estimated inter-beat interval and heart rate, which were calculated internally from the PPG signal. However, these calculations and estimations are proprietary and not described in detail, and upon visual inspection, we found discrepancies to the expected heart rate calculated from our gold-standard ECG. Therefore, we decided to employ algorithms for heart rate calculation that are already established, validated, and described in the literature. Local minima of the PPG signal were automatically detected by using the peak tracking algorithm published by Scholkmann [51]. The algorithm assumes a periodic or quasi-periodic signal that can be superimposed by low- or high-frequency noise. It searches for a local maximum within a scale *s_t_* (here a temporal scale), which is the only parameter that has to be set beforehand. The scaling parameter was empirically set to *s_t_* = 0.25 s, which is in good accordance with previous findings [52]. To avoid possible wrong detections of secondary wave peaks, the algorithm was applied to the negative PPG signal. Detections that occurred during sections of unreliable PPG signal quality were removed in a postprocessing step.

The Pan−Tompkins algorithm [53] was applied to the ECG signal using the implementation provided by Sedghamiz [54]. The Pan−Tompkins algorithm is a widely used method to detect the R peak of the QRS complex of the ECG signal and contains several filtering operations and a squaring step to better separate out R peaks from the background.

### 2.4. Derivation of the ECG and PPG HR

**Figure 3:**
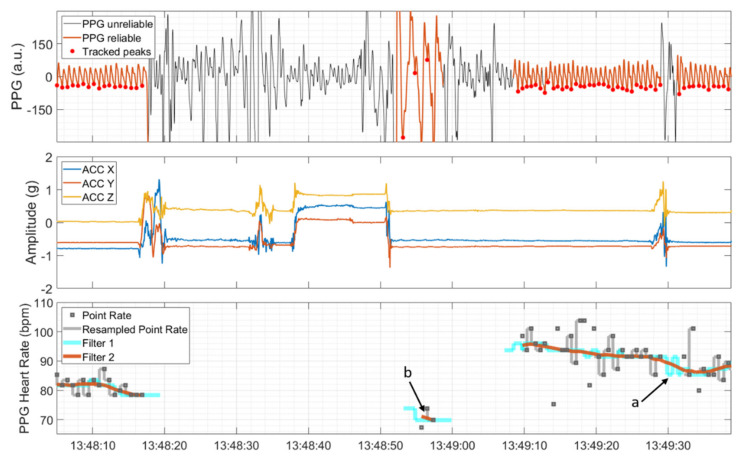
PPG signal, automatically divided into sections of reliable and unreliable quality and detected peaks (upper panel), and ACC signal showing movements at the same time (middle panel). The PPG heart rate and the associated intermediate steps; that is, resampling of the point rate, moving median (Filter 1), and moving mean (Filter 2), are depicted in the lower panel. Short duration gaps are interpolated by Filter 1 (marked by ‘a’), and isolated short PPG HR segments are removed by a postprocessing step (marked by ‘b’).

The time points of detected R peaks and the time points of local minima, detected during reliable PPG sections, were transformed to a point rate with unit of beats per minute (bpm) by taking the inverse of the difference between consecutive peaks in seconds and multiplying these values by 60 (see the upper and lower panel in Figure 3). Then, the point rate was resampled to 64 Hz and 250 Hz in correspondence with the sampling rates of the PPG signal and ECG signal, respectively. This was done by filling the time span between two consecutive point rate values with values from the latter provided that the point rate value was both inside the range from 40 to 180 bpm and changed by less than 20% compared to the previous point rate value. Otherwise, the HR algorithm produced gaps in the output. Subsequently, a symmetric two-stage low-pass filter with a length of five seconds each was used. The first moving-median filter (Filter 1 in Figure 3) was applied with the main objective of removing possible outlier sequences and interpolating gaps that lasted less than five seconds (marked by ‘a’ in Figure 3). The second moving-mean filter (Filter 2 in Figure 3) was applied to further smooth the HR. As a final postprocessing step, sections of the possibly non-continuously derivable HR that lasted less than five seconds were removed (marked by ‘b’ in Figure 3).

### 2.5. Determination of the Threshold Crossings

Two different threshold crossings were determined from ECG and PPG HR. The time period for the identification was restricted to the preictal, ictal, and postictal phase. The first threshold was defined as the first time point following the baseline interval at which the HR exceeded the baseline HR value by 20% [25,55], which is hereinafter also referred to as *PPG_20%_* and *ECG_20%_* for the PPG and ECG signals, respectively. The baseline HR value was calculated as the median HR between −60 s and −30 s relative to the EEG onset [25,29] for both the ECG and PPG signal. Prerequisite for the derivation of an averaged baseline value was a PPG signal with sufficiently high quality for a duration of at least half the baseline interval; otherwise, no baseline HR value was produced.

The second threshold was defined as the first time point following the baseline interval at which the HR exceeded 100 bpm, which are hereinafter also referred to as *PPG_>100_* and *ECG_>100_* for the PPG and ECG signal, respectively.

### 2.6. Definitions of Movement

Based on the video-EEG recordings, time intervals of clinical seizure manifestations, including epileptic movements, were annotated by a board-certified expert (NE). Time intervals with epileptic movements in the form of tonic or clonic movements, or automatisms performed with the arms or legs, either unilaterally or bilaterally, were considered relevant. Epileptic movements involving the upper or lower limbs but clinically describable neither as clonic nor tonic nor automatisms were also considered relevant and categorized as not further classifiable. Oral automatisms and eye blinking were considered as non-relevant epileptic movements. Note that seizures with hyperkinetic, myoclonic, or atonic motor manifestations were not present in this dataset.

Thus, relevant epileptic motor phenomena were based on clinical annotations and did not refer to movements actually captured by the device. In order to differentiate these movements from the clinical annotations, we investigate the ACC signal of the wearable device. For the 3D wrist acceleration signal provided by the Empatica E4 device, a time-resolved sample wise activity feature *a(t)* with units of g was calculated by summing the standard deviation *std(·)* of the 3D ACC signal in a window of length *T* [56,57]:

(2) $$a\left(t\right)=\overset{3}{\underset{i=1}{\sum }}std\;\left(ACC_{i}\left[t-\frac{T}{2},t+\frac{T}{2}\right]\right)$$

with *ACC_i_* given by the acceleration in the *i*th direction. The window length *T* was set to 1 s. If *a(t)* was above a threshold 0.05 g, the patient was assumed to perform active movements. If the value was below the threshold of 0.05 g, the patient was assumed to be at rest. The active state was further divided into epileptic and spontaneous movements as follows: Time intervals with simultaneous relevant epileptic movements were expressed as *epileptic_ACC_*. The remaining intervals were attributed to spontaneous movements.

In summary, we distinguish between five categories of movement: *relevant* and *non-relevant epileptic* (based on clinical annotations), and *epileptic_ACC_*, *spontaneous*, and *rest* (based on clinical annotations and the ACC signal).

### 2.7. Evaluation of ‘Hits’ and ‘Misses’

We describe seizures as ‘hits’ if a PPG HR threshold crossing was identified. In each of the seizures studied, it was given that at least one of the two ECG-based thresholds, that is either *ECG_20%_* or *ECG_>100_*, was determinable. Seizures are described as ‘misses’ if identification was based solely on ECG, and no threshold crossing could be identified based on the PPG signal. Note that the definition of hits and misses from information retrieval or machine learning, in the sense of true positives and false negatives, is not applicable here. Furthermore, as the work presented here investigates only peri-ictal data immediately around seizures, where tachycardia is always present in our dataset, false positives and true negatives cannot be reported as would be the case if seizure detection was investigated.

**Figure 4:**
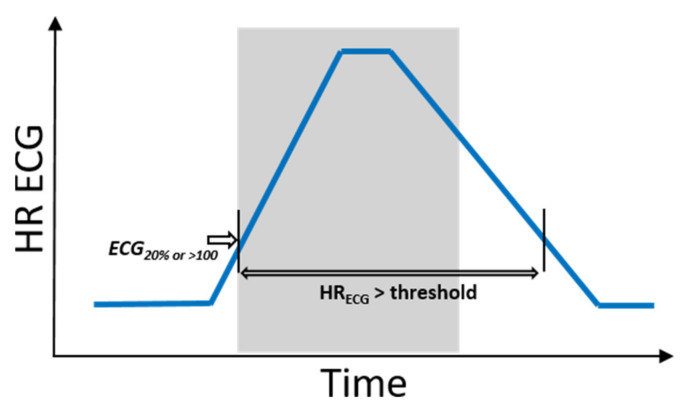
Schematic illustrations of a seizure with IT and corresponding time interval during which, based on ECG, IT is measurable. The ictal phase is marked in gray.

Hence, hits and misses were evaluated as follows: To define phases of ictal tachycardia, the time interval was determined during which the ECG HR was above the threshold: that is, either 20% above the ECG baseline HR value or beyond 100 bpm. This time interval was not necessarily restricted to the ictal phase (see Figure 4). In order to investigate the impact of movements on PPG signal quality, the activity feature *a(t)* was then evaluated by calculating the proportions of *epileptic_ACC_*, spontaneous movements, and resting phases during this time interval (see *Definitions of movement* for terminology).

## 3. Results

**Figure A1:**
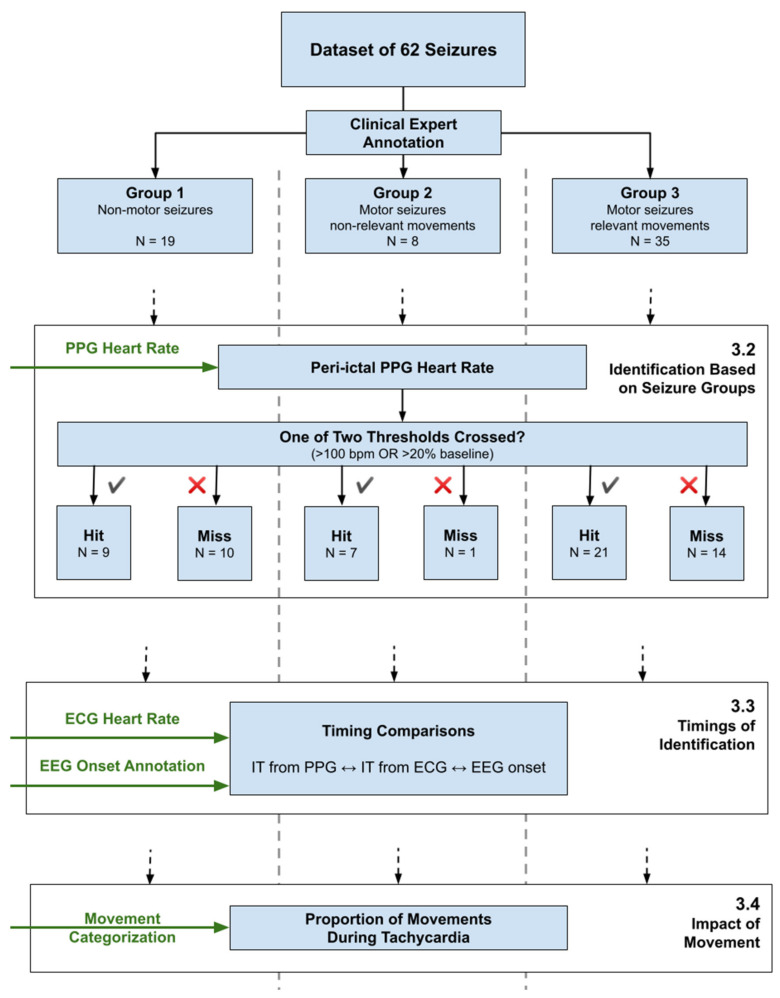
Overview of the data analysis pipeline, showing the separation of the seizure set into three groups, and the three main analysis topics, referencing Section 3.

The results are structured as follows: First, independently of the groups, the number of PPG-based identifications of IT is summarized both for *PPG_20%_* and *PPG_>100_*. In the second part, the number of group-specific identifications is given. In the third part, the timing of identification of IT, based on PPG, is compared to both ECG and EEG onset. The fourth part summarizes the impact of movements on the identification of IT. In the final section, two examples are given for a seizure that was identified (hit) and not identified (miss) by means of PPG. See also Figure A1 in the Appendix A for an overview of the analysis process.

### 3.1. Overall PPG-Based Identification

Table 1 shows that based on PPG and the determination of either *PPG_20%_* or *PPG_>100_* or both, identification of IT was possible for 37/62 seizures (60%) from 21 patients. Considered separately, the number of hits was 33/62 (53%) seizures from 17 patients for the HR increase by 20% compared to the baseline HR and 23/51 (45%) seizures from 13 patients for the HR increase beyond the threshold of 100 bpm. Based solely on the ECG HR, *ECG_20%_* was determinable for all analyzed seizures, while for 11/62 (18%) seizures, the threshold crossing of 100 bpm was either not reached (n = 8) or not considered due to a baseline HR above 100 bpm (n = 3).

### 3.2. Identification Based on Seizure Groups

The total of 62 seizures consisted of 19 non-motor seizures, where 15 contained exclusively autonomic signs and four were classified as focal impaired awareness seizures. Eight seizures had non-relevant epileptic motor activity in addition to IT, and the remaining 35 seizures showed IT as well as relevant epileptic motor activity. As summarized in Table 2, in 9/19 (47%) non-motor seizures, 7/8 (88%) seizures with non-relevant epileptic motor activity and 21/35 (60%) seizures with relevant epileptic motor activity, the identification of at least one of the two PPG-based thresholds was possible. Non-relevant epileptic movements were mostly oral automatisms (n = 7) and in one case bilateral eye blinking. The seizure of this group that was not identifiable by means of the PPG signal involved oral automatisms.

Eleven different combinations of motor manifestations (see Table A2 in the Appendix A) were determined for seizures with relevant epileptic movements, either overlapping in time or occurring sequentially. It was found that especially in seizures involving solely automatisms, IT could be determined by means of the PPG signal (hits: n = 9, misses: n = 1), where IT was identified before the onset of relevant epileptic movements in n = 6 seizures. For all other possible combinations, results were variable.

### 3.3. Timings of Identification

**Figure 5:**
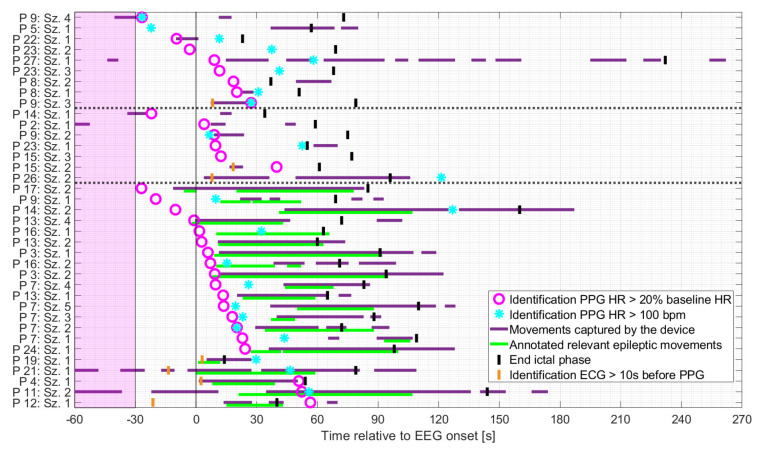
Seizures rated as hits ordered by timing of identification of IT relative to EEG onset, based on the earlier of the two PPG-based threshold crossings, and divided into non-motor seizures, seizures with non-relevant epileptic movements, and seizures with relevant epileptic movements (in this order and separated by the dashed horizontal lines). The timing of identification of the earlier of the two ECG-based threshold crossings is plotted for those cases in which identification based on PPG occurred at least 10 s later.

As shown in Figure 5, patients were mainly at rest during the baseline period and the preictal phase for all three groups. Based on the earlier of the two PPG-based thresholds, the identification of IT occurred mainly either before the onset of spontaneous movements or the initial manifestation of relevant epileptic movements, or during periods when the patient was at rest. More specifically, in seizures with relevant epileptic motor activity, the timing of the identification of IT, based on PPG, preceded the initial relevant epileptic movement in n = 14 cases (median 16.4 s), which occurred simultaneously in n = 2 cases (within in a range of 1 s) and afterwards in n = 5 cases (median 41.6 s). The earlier of the two PPG-based threshold crossings occurred mainly during the ictal phase, regardless of the seizure group. For some seizures, IT was determinable prior to EEG onset (n = 1 and n = 3 for seizures with non-relevant or relevant epileptic movements, respectively and n = 4 for non-motor seizures).

Regardless of the seizure group, the identification of *PPG_20%_* preceded that of *PPG_>100_* in 15/19 cases in which both thresholds were crossed.

**Figure 6:**
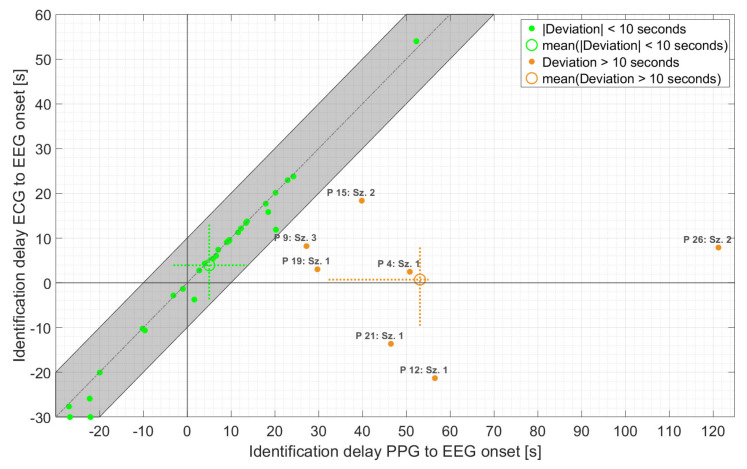
Time of identification of ECG- vs. PPG-based threshold crossings relative to EEG onset, for those seizures for which IT was found by at least one threshold crossing. The identification time refers to the earlier of the two (i.e., either HR > 20% baseline HR or HR > 100 bpm). The dashed crosses at the mean points indicate the 25th to 75th percentile.

As is evident from Figure 6, the majority of PPG-based threshold crossings were found in very good temporal agreement with those based on ECG (n = 30 with absolute deviation < 10 s), with an average delay of 5.0 s relative to EEG onset. In addition, the maximum deviation of the PPG- and ECG-based HRs at the time point of PPG-based detection ranged from 3 bpm for both non-motor and seizures with non-relevant epileptic movements to 5 bpm for seizures with relevant epileptic movements. Overall, the earlier of the two threshold crossings, based on PPG, occurred with an average delay of 14.1 s relative to EEG onset compared to 3.3 s for the earlier ECG-based threshold crossing. The difference of about 11 s was mainly caused by seven seizures. For these seven seizures, the deviation between PPG- and ECG-based threshold crossings was more than 10 s, and the averaged delay relative to EEG onset increased to 53.1 s. In all these seven seizures, movements—spontaneous or relevant epileptic or both—were present during the delay interval. Four of these seizures had relevant epileptic movements (group 3). Individually, there was considerable variation in the timing of threshold crossings relative to EEG onset for both the PPG and ECG signals.

### 3.4. Impact of Movement on PPG-Based Identification

**Figure 7:**
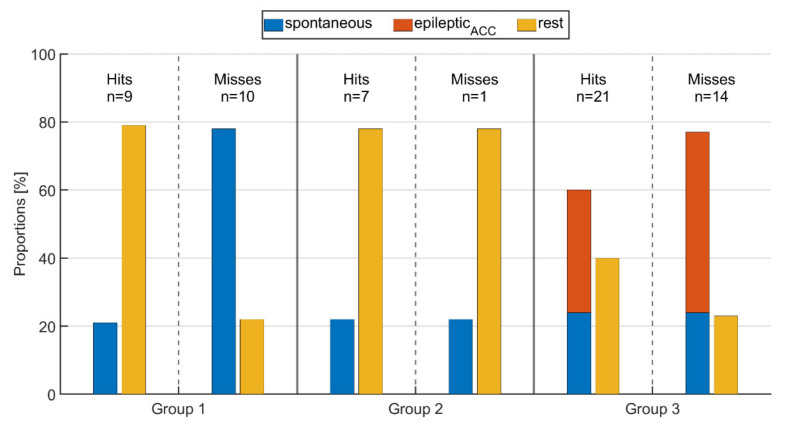
Proportion of spontaneous movements, epileptic movements (*epileptic_ACC_*), and resting phases during time intervals of (ictal or peri-ictal) tachycardia for seizures rated as either hit or miss for the respective groups.

Figure 7 shows the proportions of spontaneous, *epileptic_ACC_* movements and rest for all three seizure groups and both the cases where IT was identified and where it was not. Movement and resting phases are determined from the ACC signal of the wearable device, and specifically, epileptic movements are defined as overlaps of clinical expert labeling and ACC activity (see *Definitions of movement*). This may also replace labeled relevant epileptic movement as rest if it was not captured by the ACC signal (e.g., P.16:Sz.1 in Figure 5).

For those threshold crossings identified via PPG (hit), the average proportion of rest and movements did not change from group 1 to group 2, i.e., between seizures without epileptic movements and those with non-relevant epileptic movement such as oral automatisms. As expected, in group 3 with seizures that had relevant epileptic movement, the average fraction of rest was significantly lower than in the other groups, while the fraction of spontaneous movements was the same. Looking at those seizures where the threshold crossings were not identified by PPG (miss), group 1 had significantly higher average spontaneous motor activity during IT (78%) compared to those where IT was identified (21%). In group 2, the amount of resting phases was the same for both hits and misses (78%); however, the threshold crossing was not identified for only one seizure (see Figure 7), making a comparison between the two problematic. As for group 1, the proportions of motor activity in group 3 were higher for seizures where IT was not identified (77%) than for those where IT was identified (60%). Again, as expected, the average amount of *epileptic_ACC_* movements was higher than that of spontaneous movements in group 3. Overall, the lowest average amount of rest (22%) as well as the highest amount of movement (78%) across all groups, whether IT was identified or not, was present in group 1 for those seizures where the IT was not identified (miss). This shows the major impact of spontaneous, non-epileptic movements on the performance of PPG-based identification of tachycardia.

### 3.5. Examples

**Figure 8:**
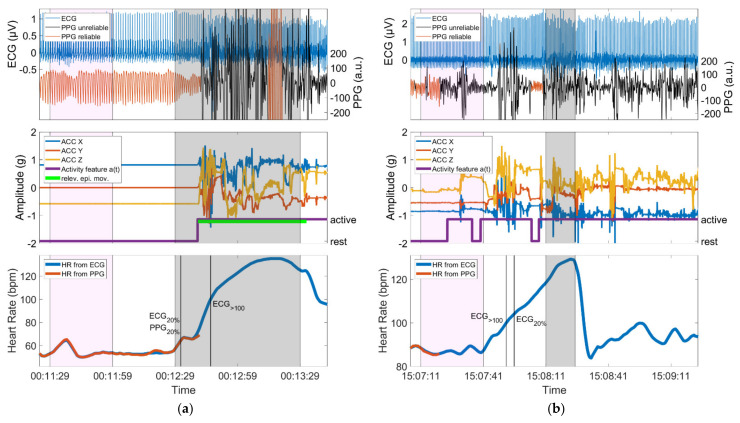
(**a**) Seizure with motor automatisms (relev. epi. mov.) performed with the left arm during the ictal phase; the device is worn on the left wrist. The identification of *PPG_20%_* occurs before the initial arm automatisms and is in very good temporal agreement with *ECG_20%_*, whereas *PPG_>100_* is not identified, as *ECG_>100_* is only crossed during the phase of epileptic movements; (**b**) Seizure with almost continuous spontaneous movements during the baseline period, the preictal, ictal and postictal phase. Identification of IT is not possible due to the spontaneous movements. In both panels, the baseline period and the ictal phase are highlighted in magenta and gray, respectively. Vertical lines mark the timing of identification of threshold crossings. Both panels show data from a period of 140 s.

In Figure 8, one example of a seizure is given with relevant epileptic movements in which an IT-threshold crossing could be identified (group 3, hits) and one example of a non-motor seizure in which the threshold crossing of IT could not be identified (group 1, misses). For each seizure, the simultaneous ECG signal is shown next to the automatically assessed PPG signal. Furthermore, the ACC signal plus the activity feature *a(t)* derived from it and the PPG- and ECG-based HR are depicted. Note that the activity feature *a(t)* is displayed in binary form, which is composed of active and rest phases (see *Definitions of movement*). The baseline period, the ictal phase, and the time points of the identifications of IT (if available) are highlighted.

## 4. Discussion

In this study, we provide (a) a comparison between PPG- and ECG-based identification of ictal tachycardia and its identification in time with regard to the EEG onset, and (b) analysis of the effect of epileptic and spontaneous movements on PPG signal quality during IT. Identification of IT by means of the wearable PPG signal was possible in 37/62 (60%) seizures and occurred, as expected, mainly when the patients were at rest.

An interesting result of this study is that PPG-based identification of ictal tachycardia was not limited to non-motor seizures. We had hypothesized that IT in non-motor seizures would be identifiable most frequently, whereas seizures with major motor components might be missed due to an insufficient PPG signal quality. We found that even in the group of seizures with relevant epileptic movements, in 60% of seizures, an IT could be identified using PPG signals. This was mostly due to the fact that the thresholds defining IT were often crossed before the onset of epileptic movements (Figure 5 and Figure 8, left). This temporal evolution of ictal HR increase followed by epileptic motor activity has been reported in previous studies [55,58,59,60]. In our sample of patients with focal epilepsy, tachycardia (based on ECG) infrequently occurred only secondary to the onset of epileptic motor activity (2/35, 6%). This indicates an early ictal epileptic involvement of the autonomic nervous system rather than secondary adaptive changes in the heart rate during the vast majority of seizures. In the dataset at hand, PPG- and ECG-based ictal tachycardia had good temporal agreement in 30/37 seizures in which IT could be identified with both methods. It is also of high interest that IT was frequently an early seizure manifestation with an average delay of 5.0 s relative to EEG onset for those seizures with good temporal agreement to ECG. The results are in line with the findings from Zijlmans [29], where the onset of IT was mostly found around electrographic seizure onset. The identification of IT during the postictal phase occurred only for seizures with epileptic movements (n = 1 and n = 2 for seizures with non-relevant or relevant epileptic movements, respectively). In all three cases, this occurred when the PPG signal quality improved after movement artifact cessation, the heart rate could be calculated again, and it was already above the specified thresholds.

In contrast, we also found evidence that movements in general may impair the PPG signal during IT identification. Especially, non-motor seizures (group 1) where the IT could not be identified were often accompanied by spontaneous movements. On the other hand, for those seizures in the same group where IT could be identified, the patient was mainly at rest (Figure 7). This indicates a strong negative effect of spontaneous movements on the ability to identify IT in non-motor seizures (Figure 8, right), which we expected to be easily identifiable. Independently, we could see a similar effect in the seizures with relevant motor phenomena (group 3), albeit to a lesser extent. Seizures in this group where the IT could not be identified had on average a higher proportion of *epileptic_ACC_* movements (53%) than those where the IT was found (36%). It stands out that in our set of seizures, those in group 3, whether the IT was found or not, often had only minor spontaneous activity in the preictal phase. However, we could not determine any correlation with seizure type for these occurrences. For these seizures, other reasons for missing the IT can be hypothesized, such as influences of external light sources on the PPG sensor or displaced sensor armbands.

A study from Vandecasteele [16], in which the same device was used, reports a sensitivity of 32% based on 47 temporal lobe seizures from 11 subjects, where the detection of IT was restricted to the detection interval lasting from 30 s before to 90 s after EEG onset. They identified motion artifacts as the main reason for failed detection, although they did not further specify them as resulting from spontaneous or epileptic activity. They use a set of rules published by De Cooman [61] to analyze their PPG signals and identify IT. However, they mention that 11 of the 47 seizures in their dataset were not associated with IT. Thus, their results should be adapted to include only seizures with IT, such that the results are comparable to the seizures analyzed in this study, encompassing only 36 seizures and resulting in an adapted sensitivity of 42%. Our approach was able to identify IT in a larger percentage of seizures from a more diverse set of patients.

Furthermore, to investigate a potential circadian preference, the Hodges−Ajne test was applied for both seizures described as hit or miss, which tests for the uniformity of circular data [62]. The timing of the day of the respective EEG onset was considered as the circular variable. The Hodges−Ajne test revealed no circadian preferences in our dataset for the identification of IT based on the PPG signal, meaning that daytime seizures could be identified as well as nocturnal seizures.

The main limitation of the work presented here is that sensitivity is not reported in combination with a false alarm rate. The identification of IT was only applied with a priori knowledge of seizures, such that no detection of seizures was carried out. In a system that directly applies this methodology, the false alarm rate would be very high due to the relatively simple requirement of crossing a threshold in the heart rate to identify a seizure. However, the purpose of this work was not to build a seizure detector but to analyze patient subgroups with different degrees of epileptic motor components, with the goal of identifying those where the detection of seizures based on ictal tachycardia by means of wearable PPG sensors is feasible. Another limitation of this study is the use of thresholds for tachycardia with fixed definitions. Individualized thresholds optimized per participant may increase the hit rate for prospective seizure detection methods, due to the dependance of the baseline heart rate on factors such as age or physical condition [28]. Note also that we specifically did not evaluate heart rate variability (HRV) features in this analysis. Due to the larger window sizes needed for their calculation and the much larger dependence on clean data without any artifacts, these HRV features are better suited for analysis based on ECG signals [21]. Here, we specifically wanted to evaluate the use of HR, which is calculated from the PPG signal, in identifying ictal tachycardia.

For future applications, the influence of motion on the PPG signal quality should also be considered in a larger temporal context. In addition, the intensity of movements might be investigated in a multi-class fashion instead of a binary activity feature. To conclude, IT identification is possible also in seizures with relevant motor phenomena because IT often precedes epileptic movements. However, spontaneous movements can impair PPG signal quality during phases of IT, which particularly impairs the detection of IT in non-motor seizures. Thus, seizures with predominant behavioral arrest may be well suited for detection of the autonomic manifestation of ictal tachycardia, whereas epileptic motor manifestations do not preclude patients from profiting from PPG signal analysis for the identification of seizures. While we have shown that the heart rate from PPG signals could have some value in increasing the sensitivity of seizure detection systems, a monomodal application of PPG data would likely result in low specificities. Rather, the results from this study must be combined into a multimodal system including, for example, both ACC and EDA signals, to have a chance at reaching a robust seizure detection.

## Figures and Tables

**Table 1:** Number of hits and misses and corresponding number of determinable ECG thresholds.

		Hits Based on PPG	Misses Based on PPG	Total ECG Thresholds
Identifications	20% increase or >100 bpm	37 (60%)	25 (40%)	62
	20% increase	33 (53%)	29 (47%)	62
	>100 bpm	23 (45%)	28 (55%)	51

**Table 2:** Number of hits and misses based on at least one of the two PPG-based thresholds and divided into non-motor seizures (group 1) and seizures with non-relevant (group 2) or relevant (group 3) epileptic movements, respectively.

	Hits Based on PPG_20%_ or PPG_>100_ 37/62	Misses Based on PPG 25/62
Group 1	9	10
Group 2	7	1
Group 3	21	14

## Appendix A

**Table A1:** Overview of patient demographics. TLE = temporal lobe epilepsy, xTLE = extratemporal lobe epilepsy.

Patient	Age	Gender	Recording Duration (Days)	Epilepsy Origin	Epilepsy Type	Number of Seizures	Seizure Duration (s) [min, max]
1	33	m	5	structural	focal (TLE)	1	101
2	32	m	5	structural	focal (TLE)	1	59
3	55	m	5	structural	focal (TLE)	5	77 [54, 94]
4	9	m	3	structural	focal (xTLE)	1	54
5	19	f	4	structural	focal (TLE)	1	57
6	35	f	5	unknown	focal (TLE)	1	83
7	27	f	5	structural	focal (TLE)	5	92 [72, 110]
8	53	f	5	structural	focal (TLE)	3	40 [32, 51]
9	14	f	4	structural	focal (xTLE)	4	74 [69, 79]
10	21	f	8	structural	focal (TLE)	5	53 [47, 61]
11	69	f	7	structural	focal (TLE)	2	153 [144, 161]
12	35	f	11	structural	focal (TLE)	1	40
13	27	f	5	structural	focal (TLE)	4	68 [60, 73]
14	26	f	6	structural	focal (TLE)	2	97 [34, 160]
15	48	m	14	structural	focal (TLE)	3	69 [61, 77]
16	68	f	6	structural	focal (TLE)	2	67 [63, 71]
17	34	f	4	unknown	focal (TLE)	2	71 [56, 85]
18	56	m	8	structural	focal (TLE)	1	116
19	16	m	3	structural	focal (TLE)	1	14
20	26	m	6	structural	focal (xTLE)	5	118 [107, 123]
21	48	m	8	structural	focal (TLE)	1	79
22	45	m	4	structural	focal (TLE)	1	23
23	41	f	8	structural	focal (TLE)	4	57 [37, 69]
24	46	f	11	structural	focal (TLE)	1	98
25	25	m	4	structural	focal (xTLE)	1	162
26	58	f	7	structural	focal (TLE)	2	76 [55, 96]
27	22	m	7	structural	focal (TLE)	1	232
28	27	f	8	structural	focal (xTLE)	1	14

**Table A2:** Number of identified and non-identified motor seizures and corresponding motor manifestations. Epileptic movements not further classifiable are referred to as ‘other’.

	Motor Type	Hits Based on Either PPG_20%_ or PPG_>100_ or Both	Misses Based on PPG
Seizures with non-relevant epileptic movements	oral automatism	6	1
	eye blinking	1	-
Seizures with relevant epileptic movements	automatism	9	1
	automatism, other	2	2
	automatism, clonic	1	1
	tonic, clonic	3	3
	tonic	2	1
	tonic, other	2	-
	to bilateral tonic-clonic	1	1
	to bilateral tonic-clonic, automatism	-	2
	to bilateral tonic-clonic, clonic, automatism	-	1
	to bilateral tonic-clonic, other	1	-
	other	-	2

## References

[1] Hoppe C., Feldmann M., Blachut B., Surges R., Elger C.E., Helmstaedter C. (2015). Novel techniques for automated seizure registration: Patients’ wants and needs. Epilepsy Behav..

[2] Bruno E., Simblett S., Lang A., Biondi A., Odoi C., Schulze-Bonhage A., Wykes T., Richardson M.P. (2018). Wearable technology in epilepsy: The views of patients, caregivers, and healthcare professionals. Epilepsy Behav..

[3] Bruno E., Biondi A., Böttcher S., Lees S., Schulze-Bonhage A., Richardson M.P. (2020). Day and night comfort and stability on the body of four wearable devices for seizure detection: A direct user-experience. Epilepsy Behav..

[4] Ulate-Campos A., Coughlin F., Gaínza-Lein M., Fernández I.S., Pearl P.L., Loddenkemper T. (2016). Automated seizure detection systems and their effectiveness for each type of seizure. Seizure.

[5] Van de Vel A., Cuppens K., Bonroy B., Milosevic M., Jansen K., Van Huffel S., Vanrumste B., Cras P., Lagae L., Ceulemans B. (2016). Non-EEG seizure detection systems and potential SUDEP prevention: State of the art: Review and update. Seizure.

[6] Halford J.J., Sperling M.R., Nair D.R., Dlugos D.J., Tatum W.O., Harvey J., French J.A., Pollard J.R., Faught E., Noe K.H. (2017). Detection of generalized tonic–clonic seizures using surface electromyographic monitoring. Epilepsia.

[7] Arends J., Thijs R.D., Gutter T., Ungureanu C., Cluitmans P., Van Dijk J., Van Andel J., Tan F., De Weerd A., Vledder B. (2018). Multimodal nocturnal seizure detection in a residential care setting A long-term prospective trial. Neurology.

[8] Poh M.-Z., Loddenkemper T., Reinsberger C., Swenson N.C., Goyal S., Madsen J.R., Picard R.W. (2012). Autonomic changes with seizures correlate with postictal EEG suppression. Neurology.

[9] Kusmakar S., Karmakar C.K., Yan B., O’Brien T.J., Muthuganapathy R., Palaniswami M. (2019). Automated Detection of Convulsive Seizures Using a Wearable Accelerometer Device. IEEE Trans. Biomed. Eng..

[10] Regalia G., Onorati F., Lai M., Caborni C., Picard R.W. (2019). Multimodal wrist-worn devices for seizure detection and advancing research: Focus on the Empatica wristbands. Epilepsy Res..

[11] Onorati F., Regalia G., Caborni C., Migliorini M., Bender D., Poh M.-Z.Z., Frazier C., Kovitch Thropp E., Mynatt E.D., Bidwell J. (2017). Multicenter clinical assessment of improved wearable multimodal convulsive seizure detectors. Epilepsia.

[12] Böttcher S., Manyakov N.V., Epitashvili N., Folarin A., Richardson M.P., Dümpelmann M., Schulze-Bonhage A., Van Laerhoven K. Using multimodal biosignal data from wearables to detect focal motor seizures in individual epilepsy patients. Proceedings of the 6th International Workshop on Sensor-based Activity Recognition and Interaction (iWOAR ’19). Association for Computing Machinery.

[13] Böttcher S., Bruno E., Manyakov N.V., Epitashvili N., Claes K., Glasstetter M., Thorpe S., Lees S., Dümpelmann M., Van Laerhoven K. (2021). Detecting Tonic-Clonic Seizures in Multimodal Biosignal Data from Wearables: Methodology Design and Validation. JMIR Mhealth Uhealth.

[14] Rukasha T., Woolley S.I., Kyriacou T., Collins T. (2020). Evaluation of Wearable Electronics for Epilepsy: A Systematic Review. Electronics.

[15] Beniczky S., Wiebe S., Jeppesen J., Tatum W.O., Brazdil M., Wang Y., Herman S.T., Ryvlin P. (2021). Automated seizure detection using wearable devices: A clinical practice guideline of the International League Against Epilepsy and the International Federation of Clinical Neurophysiology. Epilepsia.

[16] Vandecasteele K., De Cooman T., Gu Y., Cleeren E., Claes K., Van Paesschen W., Van Huffel S., Hunyadi B. (2017). Automated epileptic seizure detection based on wearable ECG and PPG in a hospital environment. Sensors.

[17] Opherk C., Coromilas J., Hirsch L.J. (2002). Heart rate and EKG changes in 102 seizures: Analysis of influencing factors. Epilepsy Res..

[18] Devinsky O. (2004). Effects of Seizures on Autonomic and Cardiovascular Function. Epilepsy Curr..

[19] Arbune A.A., Jeppesen J., Conradsen I., Ryvlin P., Beniczky S. (2020). Peri-ictal heart rate variability parameters as surrogate markers of seizure severity. Epilepsia.

[20] Beniczky S., Arbune A.A., Jeppesen J., Ryvlin P. (2020). Biomarkers of seizure severity derived from wearable devices. Epilepsia.

[21] Jeppesen J., Fuglsang-Frederiksen A., Johansen P., Christensen J., Wüstenhagen S., Tankisi H., Qerama E., Beniczky S. (2020). Seizure detection using heart rate variability: A prospective validation study. Epilepsia.

[22] Mohammadpour Touserkani F., Tamilia E., Coughlin F., Hammond S., El Atrache R., Jackson M., Bendsen-Jensen M., Kim B., Connolly J., Manganaro S. (2020). Photoplethysmographic evaluation of generalized tonic-clonic seizures. Epilepsia.

[23] Vieluf S., Reinsberger C., El Atrache R., Jackson M., Schubach S., Ufongene C., Loddenkemper T., Meisel C. (2020). Autonomic nervous system changes detected with peripheral sensors in the setting of epileptic seizures. Sci. Rep..

[24] Sevcencu C., Struijk J.J. (2010). Autonomic alterations and cardiac changes in epilepsy. Epilepsia.

[25] Hirsch M., Altenmüller D.-M., Schulze-Bonhage A. (2015). Latencies from intracranial seizure onset to ictal tachycardia: A comparison to surface EEG patterns and other clinical signs. Epilepsia.

[26] Leutmezer F., Schernthaner C., Lurger S., Pötzelberger K., Baumgartner C. (2003). Electrocardiographic changes at the onset of epileptic seizures. Epilepsia.

[27] Van Westrhenen A., De Cooman T., Lazeron R.H.C., Van Huffel S., Thijs R.D. (2019). Ictal autonomic changes as a tool for seizure detection: A systematic review. Clin. Auton. Res..

[28] Eggleston K.S., Olin B.D., Fisher R.S. (2014). Ictal tachycardia: The head-heart connection. Seizure.

[29] Zijlmans M., Flanagan D., Gotman J. (2002). Heart rate changes and ECG abnormalities during epileptic seizures: Prevalence and definition of an objective clinical sign. Epilepsia.

[30] Di Gennaro G., Quarato P.P., Sebastiano F., Esposito V., Onorati P., Grammaldo L.G., Meldolesi G.N., Mascia A., Falco C., Scoppetta C. (2004). Ictal heart rate increase precedes EEG discharge in drug-resistant mesial temporal lobe seizures. Clin. Neurophysiol..

[31] Jansen K., Varon C., Van Huffel S., Lagae L. (2013). Peri-ictal ECG changes in childhood epilepsy: Implications for detection systems. Epilepsy Behav..

[32] Bruno E., Biondi A., Richardson M.P. (2018). Pre-ictal heart rate changes: A systematic review and meta-analysis. Seizure.

[33] Allen J. (2007). Photoplethysmography and its application in clinical physiological measurement. Physiol. Meas..

[34] Zhang L., Pan J., Zhang Z., Wu H., Yao N., Cai D., Xu Y., Zhang J., Sun G., Wang L. (2020). Ultrasensitive skin-like wearable optical sensors based on glass micro/nanofibers. Opto-Electron. Adv..

[35] Xu K., Fujita Y., Lu Y., Honda S., Shiomi M., Arie T., Akita S., Takei K. (2021). A Wearable Body Condition Sensor System with Wireless Feedback Alarm Functions. Adv. Mater..

[36] Taylor S., Jaques N., Chen W., Fedor S., Sano A., Picard R. Automatic identification of artifacts in electrodermal activity data. Proceedings of the 37th Annual International Conference of the IEEE Engineering in Medicine and Biology Society (EMBC).

[37] Bent B., Goldstein B.A., Kibbe W.A., Dunn J.P. (2020). Investigating sources of inaccuracy in wearable optical heart rate sensors. Npj Digit. Med..

[38] Bruno E., Böttcher S., Viana P.F., Amengual-Gual M., Joseph B., Epitashvili N., Dümpelmann M., Glasstetter M., Biondi A., Laerhoven K. (2021). Wearable devices for seizure detection: Practical experiences and recommendations from the Wearables for Epilepsy And Research (WEAR) International Study Group. Epilepsia.

[39] Elgendi M. (2012). On the Analysis of Fingertip Photoplethysmogram Signals. Curr. Cardiol. Rev..

[40] Krishnan R., Natarajan B., Warren S. (2010). Two-stage approach for detection and reduction of motion artifacts in photoplethysmographic data. IEEE Trans. Biomed. Eng..

[41] Yousefi R., Nourani M., Ostadabbas S., Panahi I. (2014). A motion-tolerant adaptive algorithm for wearable photoplethysmographic biosensors. IEEE J. Biomed. Health Inform..

[42] Zhang Z., Pi Z., Liu B. (2015). TROIKA: A general framework for heart rate monitoring using wrist-type photoplethysmographic signals during intensive physical exercise. IEEE Trans. Biomed. Eng..

[43] Zhang Z. (2015). Photoplethysmography-based heart rate monitoring in physical activities via joint sparse spectrum reconstruction. IEEE Trans. Biomed. Eng..

[44] Ye Y., Cheng Y., He W., Hou M., Zhang Z. (2016). Combining Nonlinear Adaptive Filtering and Signal Decomposition for Motion Artifact Removal in Wearable Photoplethysmography. IEEE Sens. J..

[45] Karlen W., Raman S., Ansermino J.M., Dumont G.A. (2013). Multiparameter respiratory rate estimation from the photoplethysmogram. IEEE Trans. Biomed. Eng..

[46] Orphanidou C., Bonnici T., Charlton P., Clifton D., Vallance D., Tarassenko L. (2015). Signal-quality indices for the electrocardiogram and photoplethysmogram: Derivation and applications to wireless monitoring. IEEE J. Biomed. Health Inform..

[47] Elgendi M. (2016). Optimal signal quality index for photoplethysmogram signals. Bioengineering.

[48] Selvaraj N., Mendelson Y., Shelley K.H., Silverman D.G., Chon K.H. Statistical approach for the detection of motion/noise artifacts in Photoplethysmogram. Proceedings of the Annual International Conference of the IEEE Engineering in Medicine and Biology Society (EMBC).

[49] Nasseri M., Nurse E., Glasstetter M., Böttcher S., Gregg N.M., Laks Nandakumar A., Joseph B., Pal Attia T., Viana P.F., Bruno E. (2020). Signal quality and patient experience with wearable devices for epilepsy management. Epilepsia.

[50] Harris F.J. (1978). On the Use of Windows for Harmonic Analysis with the Discrete Fourier Transform. Proc. IEEE.

[51] Scholkmann F., Boss J., Wolf M. (2012). An efficient algorithm for automatic peak detection in noisy periodic and quasi-periodic signals. Algorithms.

[52] Wójcikowski M., Pankiewicz B. (2020). Photoplethysmographic time-domain heart rate measurement algorithm for resource-constrained wearable devices and its implementation. Sensors.

[53] Pan J., Tompkins W.J. (1985). A Real-Time QRS Detection Algorithm. IEEE Trans. Biomed. Eng..

[54] Sedghamiz H. Matlab Implementation of Pan Tompkins ECG QRS Detector. **2014**, 1–3. https://www.researchgate.net/publication/313673153_Matlab_Implementation_of_Pan_Tompkins_ECG_QRS_detector.

[55] Bruno E., Biondi A., Richardson M.P., On behalf of the RADAR-CNS Consortium (2021). Digital semiology and time-evolution pattern of bio-signals in focal onset motor seizures. Seizure.

[56] Parkka J., Ermes M., Korpipaa P., Mantyjarvi J., Peltola J., Korhonen I. (2006). Activity Classification Using Realistic Data From Wearable Sensors. IEEE Trans. Inf. Technol. Biomed..

[57] Bruno E., Böttcher S., Biondi A., Epitashvili N., Manyakov N.V., Lees S., Schulze-Bonhage A., Richardson M.P. (2020). Post-ictal accelerometer silence as a marker of post-ictal immobility. Epilepsia.

[58] Guggisberg A.G., Hess C.W., Mathis J. (2007). The significance of the sympathetic nervous system in the pathophysiology of periodic leg movements in sleep. Sleep.

[59] Calandra-Buonaura G., Toschi N., Provini F., Corazza I., Bisulli F., Barletta G., Vandi S., Montagna P., Guerrisi M., Tinuper P. (2012). Physiologic autonomic arousal heralds motor manifestations of seizures in nocturnal frontal lobe epilepsy: Implications for pathophysiology. Sleep Med..

[60] San Antonio-Arce V., Schulze-Bonhage A. (2015). Ictal tachycardia in patients with hypothalamic hamartoma. J. Neurol. Neurosurg. Psychiatry.

[61] De Cooman T., Varon C., Van De Vel A., Ceulemans B., Lagae L., Van Huffel S. Semi-supervised one-class transfer learning for heart rate based epileptic seizure detection. Proceedings of the Computing in Cardiology (CinC).

[62] Berens P. (2009). CircStat: A MATLAB Toolbox for Circular Statistics. J. Stat. Softw..

